# Supplementary material for: High-resolution single-photon imaging with physics-informed deep learning
Source: Nat Commun. 2023 Sep 22;14:5902. doi: 10.1038/s41467-023-41597-9 (PMC10516985; doi:10.1038/s41467-023-41597-9)
Supplement: Supplementary file 1 — Supplementary Information [file 41467_2023_41597_MOESM1_ESM.pdf]

Supplementary Information for High-resolution Large-scale single-photon imaging with physics-informed deep learning

Liheng Bian[1,2,#,*], Haoze Song[1,#], Lintao Peng[1], Xuyang Chang[1], Xi Yang[3], Roarke Horstmeyer[3], Lin Ye[4], Chunli Zhu[1], Tong Qin[1], Dezhi Zheng[1,2], and Jun Zhang[1,*]

[1] *MIIT Key Laboratory of Complex-field Intelligent Sensing, Beijing Institute of Technology, Beijing 100081, China*

[2] *Yangtze Delta Region Academy of Beijing Institute of Technology (Jiaxing), Jiaxing 314019, China*

[3] *Department of Biomedical Engineering, Duke University, Durham, North Carolina 27708, USA*

[4] *School of Materials Science and Engineering, Beijing Institute of Technology, Beijing, 100081, China*

These authors contribute equally to this work.

[*] *bian@bit.edu.cn*

[*] *zhjun@bit.edu.cn*

Supplementary Note 1 - Details of calibrated noise parameters.

As stated in the Methods section, we established the multi-source physical noise model of SPAD arrays as

$$\mathrm{N} = \mathrm{N}_{\mathrm{shot}} + \mathrm{N}_{\mathrm{fp}} + \mathrm{N}_{\mathrm{dcr}} + \mathrm{N}_{\mathrm{ap}} + \mathrm{N}_{\mathrm{ct}} + \mathrm{N}_{\mathrm{dt}}. \quad (1)$$

The negative influence of $\mathrm{N}_{\mathrm{shot}}$ (shot noise), N_{fp} (fixed-pattern noise) and N_{dt} (dead-time noise) is tackled by either data-driven processing, employing manufacture parameters or adjusting hardware settings. The model parameters that are required to calibrate include the expectation of N_{dcr} (dark-count-rate noise), the fixed probability p_{ap} of N_{ap} (after-pulsing noise), and the expectation of N_{ct} (crosstalk noise). We presented the calibrated noise maps of our employed SPAD array (MPD-SPC3) in Supplementary Figure 1. We used the calibrated parameters to synthesize the large-scale single-photon image dataset for subsequent network training.

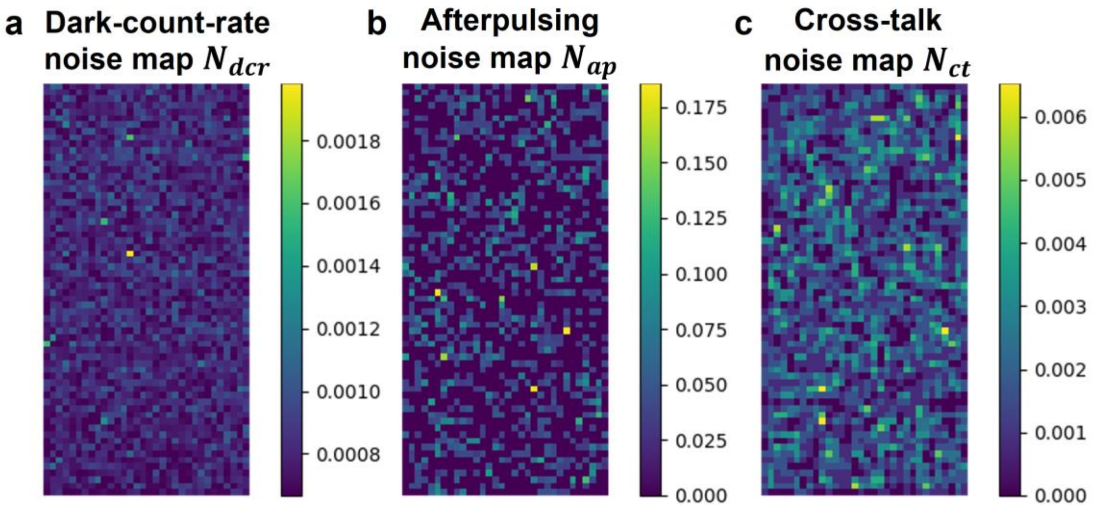

Supplementary Figure 1: The calibrated multi-source noise maps of the employed SPAD array (MPD-SPC3). (a) The dark-count-rate noise map N_{dcr}. (b) The after-pulsing noise map N_{ap}. (c) The crosstalk noise map N_{ct}.

Supplementary Note 2 - Details of the collected image datasets.

We collect two single-photon image datasets for deep learning. The first image dataset was real-acquired using our built optical setup, in which we employed the MPD-SPC3 SPAD array for single-photon detection. The targets for imaging were categorized into 3 kinds, including art, nature objects, and science & technology. Each category consists of 3 classes, and each class contains 10 scenes. We collected 10 different bit depth for each scene, with the laser power set to be 10mW, 20mW and 40mW. We provide the .mat raw data and tiff format image for each setting of the dataset. The dataset was employed to calibrate the parameters of the multi-source physical noise model.

The second single-photon image dataset was synthesized using the calibrated noise parameters, with 17250 high-resolution images (resized to 512 × 512 pixels) selecting from the public Pascal VOC2007[1] and Pascal VOC2012[2] datasets. Each high-resolution was down-sampled to different low resolution of 32×32, 64×64, 128×128, and 256×256. The simulated single-photon detection noise was added on the 32×32 low-resolution images as network inputs, with the high-resolution images serving as outputs. Similar to the realistic dataset, each scene includes images of 10 different bit depth and at 3 different illumination flux.

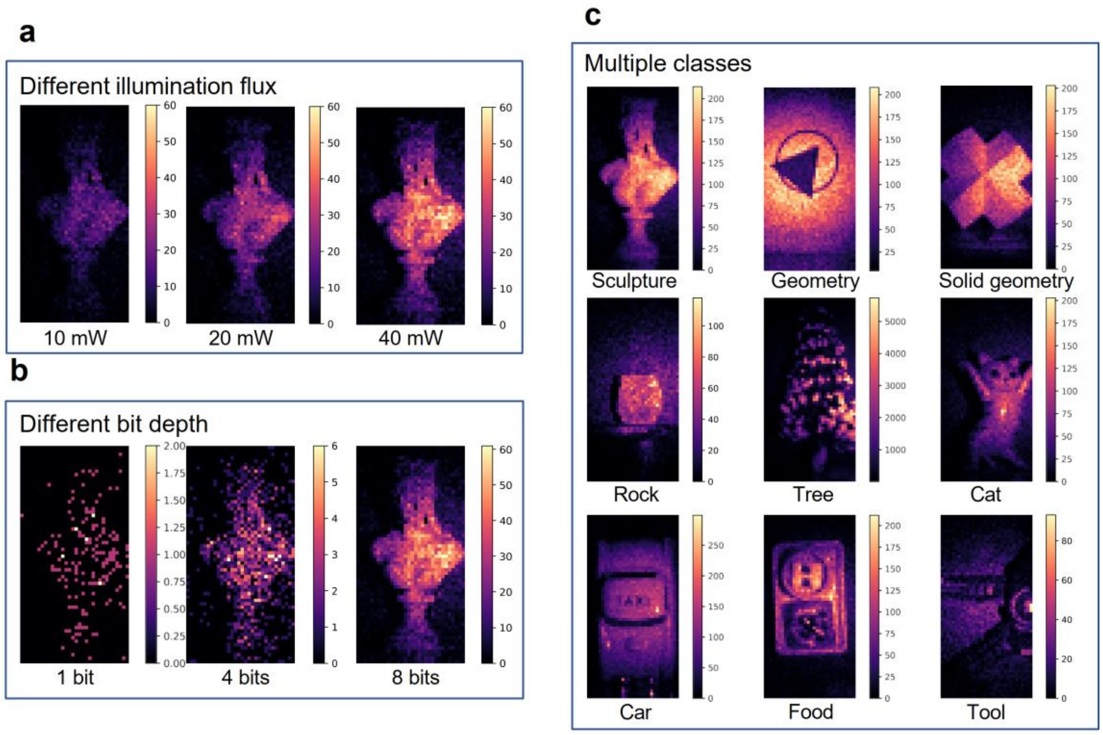

Supplementary Figure 2: The illustration of the real-acquired single-photon image dataset for noise calibration. (a) Images acquired under 3 different lighting conditions of 10mW, 20mW and 40mW. (b) Each image contains different bit depth ranging from 1 bit to 10 bits. (c) There are 9 classes of targets in the dataset in total.

Supplementary Note 3 - Details of the shallow feature extraction module and image reconstruction module in the network.

We designed a transformer network for single-photon imaging enhancement. The network consists of three modules, including the shallow feature extraction module, the deep feature fusion module, and the image reconstruction module. Here, we show the structure details of the shallow feature extraction module and image reconstruction module in Supplementary Table 1. The shallow feature extraction module mainly serves to extract the shallow features of the image and increase the number of channels from 3 to 96. The image reconstruction module mainly serves to improve image resolution and output a high-fidelity image.

Supplementary Table 1: Structure Details of the Shallow Feature Extraction Module and Image Reconstruction Module in the Reported Network.

Feature Extraction	Output size
Conv(1,96,3,1,1)	32*32*96
Conv(96,96,3,1,1)	32*32*96
Conv(96,96,3,1,1)	32*32*96
**Image Reconstruction**	**Output size**
Conv(96,24,3,1,1)	32*32*24
LeakyRelu(24)	32*32*24
Conv(24,24,3,1,1)	32*32*24
LeakyRelu(24)	32*32*24
Conv(24,64,3,1,1)	32*32*64
LeakyRelu(64)	32*32*64
upsample(4x,64,4)	128*128*4
Conv(4,1,3,1,1) $\rightarrow$ output	128*128*1

Supplementary Note 4 - Details of network training.

We implemented the reported gated-fusion transformer network on Ubuntu20 operating system using Python and Pytorch framework, and trained 1000 epochs using Adam optimization solver on NVIDIA RTX3090 graphics card. The batchsize was set to be 24. The learning rate was initialized to be 0.0003, and was decreased by 10% in every 100 epochs. We set the default values of β_1 and β_2 to 0.5 and 0.999, respectively. The weight decay parameter was set to be 0.00005. In the large-scale synthetic image dataset, we randomly selected 505 image pairs as the testing set. The remaining 16745 image pairs were used as the training set.

Supplementary Note 5 - More enhancement results on real-acquired images.

We provide more enhancement results on real-acquired images, to validate the effectiveness of the reported high-fidelity single-photon imaging technique. The experiment results on macroscopic images are presented in Supplementary Figure 3, and that on microscopic images are shown in Supplementary Figure 4.

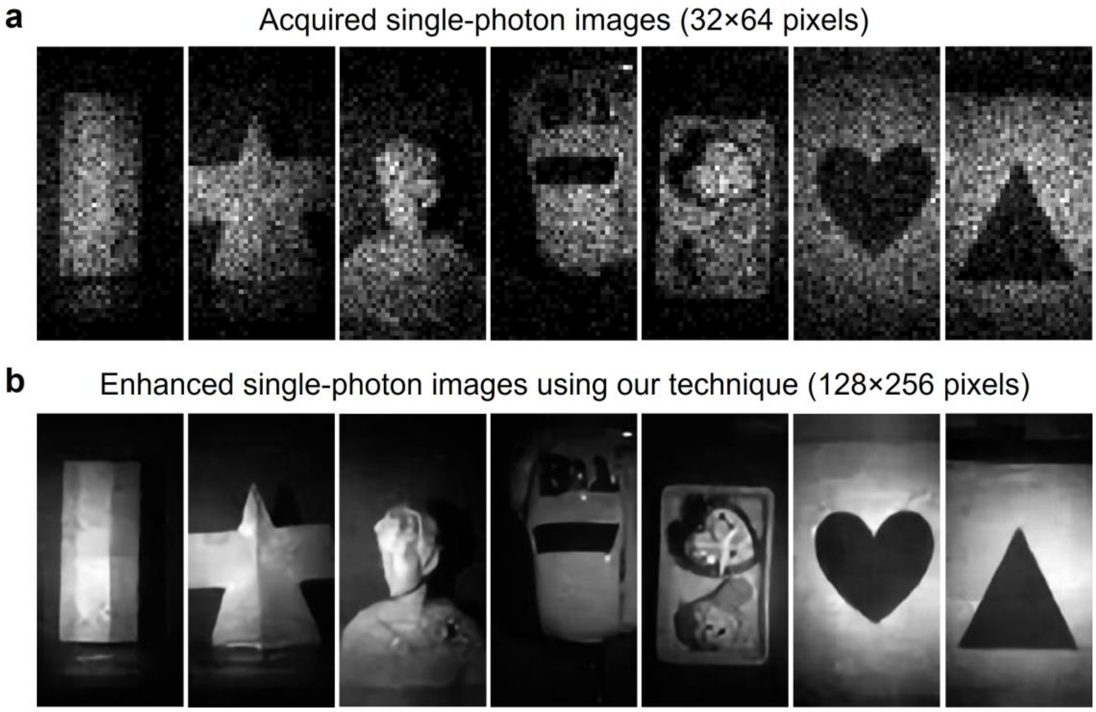

Supplementary Figure 3: Enhancement results on macroscopic single-photon images. (a) The acquired single-photon images using the optical system shown in Supplementary Figure 2(a). (b) The enhancement results using the reported neural network. After using the reported enhancement technique, the target details of such as face sculpture and different shapes are well resolved.

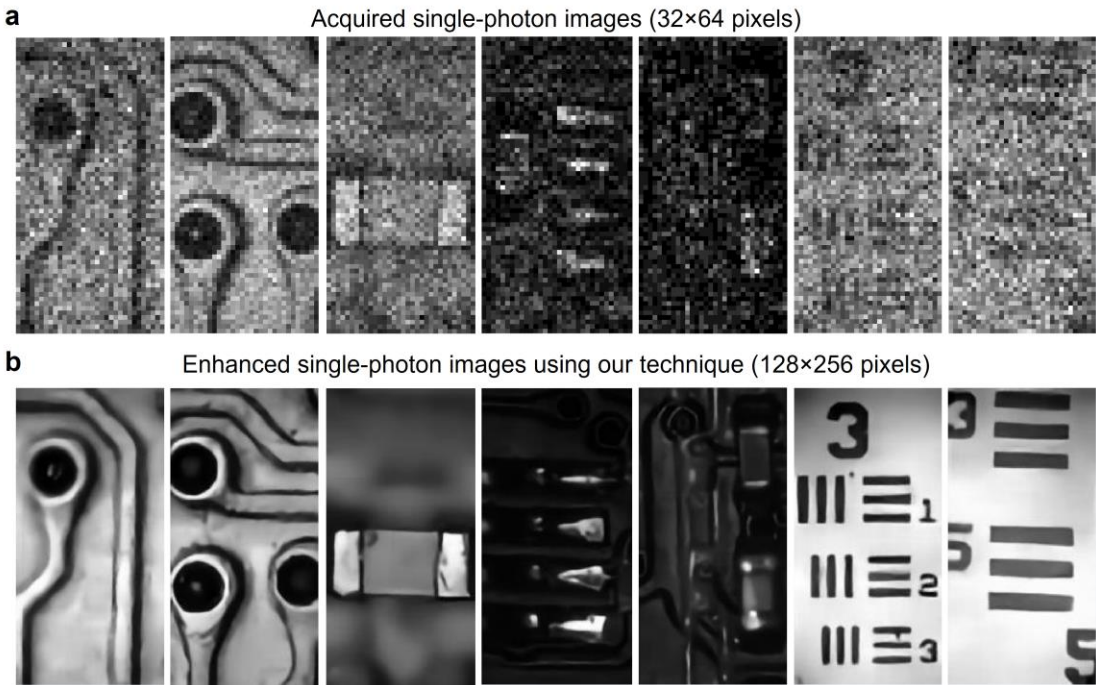

Supplementary Figure 4: Enhancement results on microscopic single-photon images including a printed circuit board and a USAF resolution target. (a) The acquired single-photon images using the optical system shown in Supplementary Figure 2(d). The imaging resolution of direct acquisition is far from revealing key details. (b) The enhancement results using the reported neural network. We can clearly resolve the details of electronic circuit and resistance components. Besides, the resolution details that can not be resolved by direct single-photon acquisition are well presented using the reported technique.

Supplementary Note 6 - More microfluidic inspection experiment results.

As stated in the Results section, we built a microfluidic inspection setup using single-photon detection, as shown in Supplementary Figure 3 (a). Here, we present more imaging results of the microfluidic inspection experimental for demonstration.

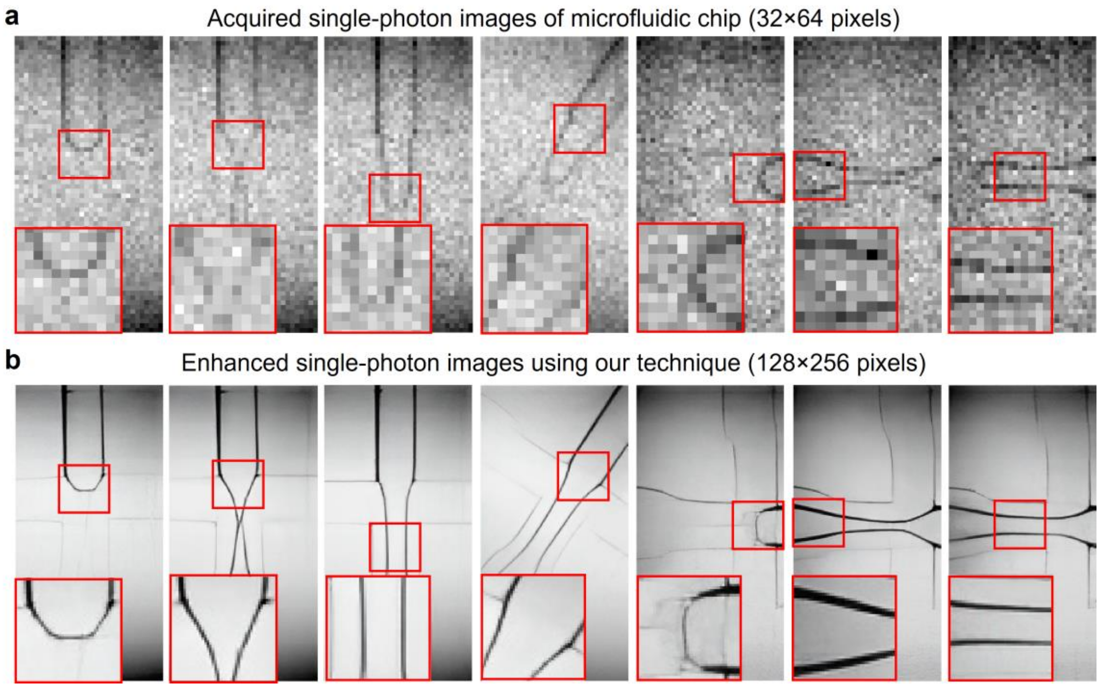

Supplementary Figure 5: The microfluidic inspection experiment results. (a) The acquired single-photon images of microfluidic chip. We can hardly resolve the micro channel structure. (b) The enhancement results using the reported technique. After high-fidelity enhancement, we can clearly resolve the channel details, which facilitate studying fluid flow and micro-droplet change process.

Supplementary Note 7 - Details of the dataset synthesis process.

Adding the multiple-noise model into synthetic images includes the following procedures.

1. We first calibrate the noise model. The noise formation model of dark-field images is

$$\mathrm{I_{dark}}(x,y,n) = \mathrm{N_{dcr}}(x,y,n) + \mathrm{p_{ap}}(x,y)\mathrm{I_{dark}}(x,y,n-1) + \mathrm{p_{ct}}(x,y)U\big(\mathrm{I_{dark}}(x,y,n)\big). \quad (2)$$

We could extract the noise maps of afterpulsing noise $\mathrm{p_{ap}}$, and crosstalk noise $\mathrm{p_{ct}}$ according to their generation mechanism. The probability of afterpulsing noise is calculated as :

$$p_{ap}(x,y) = \frac{\sum_{\mathrm{n}} \mathrm{I_{ap}}(x,y,n)}{\sum_{\mathrm{n}} \left(\mathrm{I_{dark}}(x,y,n) - \mathrm{I_{ap}}(x,y,n) - \mathrm{I_{ct}}(x,y,n)\right)}. \quad (3)$$

The probability of crosstalk noise is calculated as:

$$\mathrm{p_{ct}}(x,y) = \frac{\sum_{n} I_{ct}(x,y,n)}{\sum_{n} \left(\mathrm{I_{dark}}(x,y,n) - \mathrm{I_{ap}}(x,y,n) - \mathrm{I_{ct}}(x,y,n)\right)}. \quad (4)$$

We then calibrate the dark count noise map as

$$\mathrm{N_{dcr}}(x,y) = \frac{\sum_{\mathrm{n}} \left(\mathrm{I_{dark}}\,(x,y,n) - \mathrm{I_{ap}}(x,y,n) - \mathrm{I_{ct}}(x,y,n)\right)}{\mathrm{n_{total}}}. \quad (5)$$

In the calibration, we mainly calibrate the parameters p_{ap}, p_{ct} and N_{dcr}.

2. We then add the calibrated noise model to synthetic images following these five steps.

(1) We create a noise-free image from public datasets and normalize it to the range [0, max_photon_number × laser_power]. The max_photon_number is a typical constant corresponding to the maximum photon number of the scenes at limited photon scenarios. In this work, we use 1000 as the max_photon_number and set the laser power to 10mW, 20mW, and 40mW as the same as the experiments.

(2) For each calibrated noise source, we incorporate it into the noise-free synthetic image. Specifically, we overlay the noise model on the noise-free synthetic image on a per-pixel basis according to the calibrated parameters. In each loop, we generate a random variable R1 for each different judgment. p(Poisson) is the first noise probability

in the noise model. If R1 is less than p(Poisson), we assume the pixel detected a photon at that time, so we set the pixel value to 1.

(3) We then generate another random variable R2 and compare it to the parameter p_{ap}(afterpulsing noise probability, second noise in the noise model). If the random variable R2 is less than p_{ap} and the previous frame pixel value at the same pixel is 1, it means an afterpulsing event was detected, and we set the pixel value to 1 regardless of whether there was a detected photon before.

(4) We generate a different random variable R3 and compare It with the calibrated parameter p_{ct} (crosstalk noise probability, third noise in the noise model). If the random variable R3 is less than p_{ct} and the pixel value at the neighboring pixels is 1, it means that we detected a crosstalk event. We set the pixel value to 1 regardless of whether a photon was detected before.

(5) We sum the 1-bit frames on the time axis to obtain a multi-bit grayscale image. To account for the dark count effect, we add the Poisson random map with the expected value being the dark count rate map we calibrated. This approach enables the stacking of different noise models while controlling the noise level.

Finally, we add the calibrated multiple noise model to obtain the final synthetic image with comprehensive noise characteristics from the public dataset. The above steps are summarized in the following Supplementary Algorithm 1.

SUPPLEMENTARY ALGORITHM 1. ADD CALIBRATED NOISED MODEL TO SYNTHETIC IMAGES

Input: a zero matrix *I,* the pixel index i, the time index t, a random variable R that is generated differently each time, the p(Poisson), the neighbor region around pixel I U(i, t) , and probabilities p(afterpulsing), and p(crosstalk) that we have calibrated, we can define N_{dcr} as the Poisson random variable with an expectation value equal to the .mat file we have calibrated.

For *i =1, 2, ..., N do*

  For *t = 1, 2, ...T do*

    If *R1<p(Poisson)*

      I (i, t) = 1

    If *R2<p(afterpulsing) & I (i, t-1) ==1*

      I (i, t) = 1

    If *R3<p(crosstalk) & U (i, t) ==1*

      I (i, t) = 1

    Summarize I in t axis

$S = I+D$

Return *synthetic image S*

Supplementary Note 8 - SPAD arrays' sampling/ISP scheme and simulation strategy.

To demonstrate the uniqueness of the sampling/ISP scheme of SPAD imaging, we first analyze the sampling/ISP differences between SPAD and CMOS, and then describe the strategy for simulating the sampling/ISP scheme of SPAD arrays as follows.

1. **The sampling/ISP differences between SPAD and CMOS**

Compared to CMOS sensors, SPAD arrays have unique sampling schemes, including direct detection mode, gated mode, and time-correlated single photon counting (TCSPC) mode. In our experiments, we employ the direct detection mode, where each pixel counts photons within a defined integration time. Due to the avalanche circuit, SPAD arrays can detect at most one photon during the integration time. In addition, noise sources in SPAD arrays differ from those in CMOS sensors, as they include signal-dependent shot noise from photon incidence, fixed-pattern noise, dark count rate, afterpulsing and crosstalk noise from electron avalanche, and deadtime noise from circuit quenching.

The ISP for CMOS sensors typically involves noise reduction, color correction, white balance, gamma correction, and contrast enhancement. Differently, SPAD arrays generate raw data as a series of 1-bit images, with each image represented as a 64×32 matrix. In our experiments, the pixel value is either 0 or 1 at the 20ns integration time. The primary distinction between the ISP of CMOS sensors and SPAD arrays concerns the bit depth of the image. For CMOS sensors, the output image after ISP is either 8-bit or another value, depending on the analog-to-digital converter's (ADC) bit depth. For SPAD arrays, the bit depth depends on the total number of frames in the raw data.

2. Strategy for the simulation of SPAD arrays' sampling/ISP scheme

For the sampling scheme of SPAD arrays, we employ a direct detection model as

$$I(i,j,k) = f(i*\Delta x, j*\Delta y, p*\Delta t)$$

Considering the unique avalanche circuit of SPAD arrays that detects at most one photon during the integration time, we apply the Poisson distribution with probability p(Poisson) = 1-p(k=0), where $p(k) = \frac{e^{-\chi}\chi^k}{k!}$. This method considers the intrinsic properties of SPADs and their influence on image synthesis.

For the ISP scheme, raw data is represented as a series of 1-bit images, which

differs from the 8-bit or 16-bit images of CMOS or CCD sensors. Therefore, when synthesizing images, we set the integration time to 20 ns, resulting in each frame pixel value being either 0 or 1. To generate an n-bit SPAD image, we add 2^n continuous frames from the raw data. For the synthetic image, we follow the same process as the physics-based procedure for the n-bit image and normalize it to a range of [0,1] to be used as input of the network.

Supplementary Note 9 - Transfer learning for further enhancement.

When tackle the experiments in which the target scenes are different from that of the training dataset, one can use transfer learning to further fine-tune the pre-trained model for satisfying enhancement performance. Fine-tuning a pre-trained model requires only a small number of target scene training pairs[3].

To demonstrate that the reported Gated Fusion Transformer network can be effectively transferred to other experiment scenarios, we conducted an experiment on the microscopic white blood cell dataset, which was not included in our synthesized training dataset. In the experiment, we first synthesized noisy white blood cell microscopic images using the calibrated noise model, and then fed them directly into the pre-trained model for image enhancement. In addition, an additional fine-tuning dataset containing 50 pairs of white blood cell images was produced to fine-tune the pre-trained model. Finally, we used the fine-tuned network to enhance the noisy white blood cell images in the testing set. We present the visualization results in Supplementary Figure 6. We can see that the reported pre-trained model is able to achieve satisfactory enhancement performance after fine-tuning with a small amount of training data.

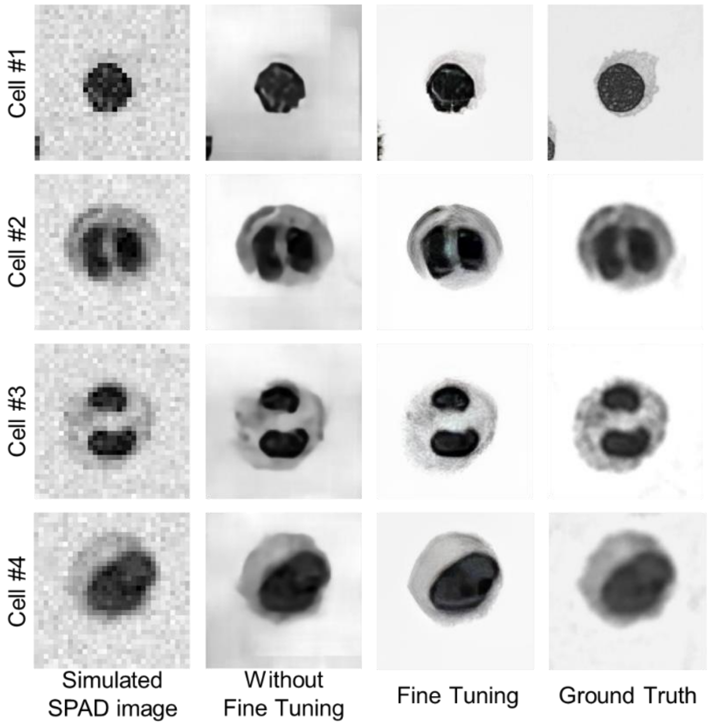

Supplementary Figure 6：Visualized image enhancement results of simulated SPAD microscopic white blood cell with pre-trained models and fine-tuned models. The pre-trained model can achieve satisfactory SPAD image enhancement results after fine-tuning.

Supplementary Note 10 - The network structure details.

1. Overall architecture

To achieve the end-to-end SPAD camera image enhancement task, we designed a novel Gated Fusion Transformer network based on the Swin Transformer structure. This network consists of three main modules: shallow feature extraction module, deep feature fusion module and image reconstruction module.

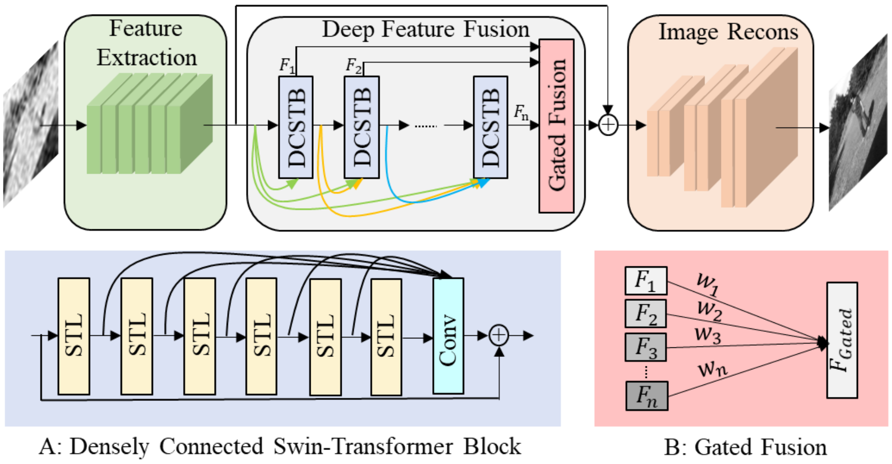

Supplementary Figure 7： The detailed structure of the Gated-Fusion Transformer network

Shallow Feature Extraction: Given a low-quality image $\mathrm{I}_{\mathrm{LQ}} \in \mathbb{R}^{h*w*c_{in}}$ (h, w and c_{in} are the image's height, width and channel number), we use the shallow feature extractor $\mathrm{H}_{\mathrm{FE}}(*)$ to explore its low-frequency features $\mathrm{F}_0 \in \mathbb{R}^{h*w*c}$ as

$$\mathrm{F}_0 = \mathrm{H}_{\mathrm{FE}}(\mathrm{I}_{\mathrm{LQ}}). \tag{6}$$

This module is composed of convolution, batch normalization and activation layers . The convolution layers are applied for preliminary visual processing, providing a simple way to map the input image space to a higher-dimensional feature space. Besides the following deep fusion operation, the output of this module is also linked to the final image reconstruction module, so that the target's low-frequency information can be well preserved in final reconstruction.

Deep Feature Fusion: Next, we use several densely connected Swin-Transformer blocks (DCSTB) to extract different levels of medium-frequency and high-frequency features $\mathrm{F}_i \in \mathbb{R}^{h*w*c} (i = 1,2, \ldots, n)$ from F_0, denoted as:

$$\mathrm{F}_i = \mathrm{H}_{\mathrm{DCSTB}}(\mathrm{F}_0, \mathrm{F}_1, \ldots, \mathrm{F}_{i-1}), \tag{7}$$

where $\mathrm{H}_{\mathrm{DCSTB}}$ represents the i_{th} DCSTB operation. Compared to the onventional convolution blocks in such as U-net, the transformer-structure blocks realizes spatial-variable convolution that helps pay more attentions to the regions of fine details and interests. Consequently, such blocks help recover more high-frequency information that is beneficial to enhancing imaging resolution.

The last layer of the deep feature fusion module is the Gated Fusion layer, which fuses the outputs of different DCSTB operations with adaptively different weights. The process can be described as

$$\mathrm{F_{DF}} = \mathrm{H_{GATE}}(\mathrm{F_1}, \mathrm{F_2}, \dots, \mathrm{F_n}) = \mathrm{w_1 F_1} + \mathrm{w_2 F_2} + \cdots + \mathrm{w_n F_n}, \tag{8}$$

where F_{DF} represents the multi-level deep fusion features output by the gated fusion layer, and w_n represents the weight parameters during gated fusion for different levels of feature, which are adaptively adjusted through backpropagation during network training. Such a module structure is conducive to deep mining of different levels of medium-frequency and high-frequency information, which prevents losing long-term memory as network deepens and enhances local details. And the densely connected structure in deep feature fusion module helps to preserve and compensate key medium and high frequency image signals, and ensures Maximum information flow between different modules.

Image Reconstruction: We retrieve high-quality single-photon images by aggregating shallow features and multi-level deep fusion features. The operation is described as

$$\mathrm{I_R} = \mathrm{H_{REC}}(\mathrm{F_0} + \mathrm{F_{DF}}), \tag{9}$$

The shallow features F_0 are mainly low-frequency image features, while the multi-level deep fusion features F_{DF} focus on recovering lost medium-frequency and high-frequency features. Benefiting from the long-term skip connections, the Gated Fusion Transformer network can effectively transmit different-frequency information to final high-quality reconstruction. Different from the state-of-the-art SwinIR network that lacks the densely connected gated fusion structure, the reported network helps preserve and compensate the key medium and high-frequency feature signals, and enriches image's local details. In addition, sub-pixel convolution is applied in the reconstruction block to further upsample the feature map for single-photon super resolution.

2. Densely Connected Swin-Transformer Block（DCSTB）

The DCSTB module consists of K Swin-Transformer Layers (STL) and a convolutional block. Given that the input of the i_{th} DCSTB is $F_i^0 \in \mathbb{R}^{h*w*c_{in}} (i = 1,2, \dots, n)$, we first use K-layers of STL to extract feature sequences $F_i^j \in \mathbb{R}^{hw*c} (i = 1,2, \dots, n; j = 1,2 \dots . k)$. Assuming that the input of the j_{th} STL is F_i^{j-1}, the calculation formula of its output is:

$$\mathrm{F_i^j} = \mathrm{H_i^{STL_j}}(\mathrm{F_i^{j-1}}). \tag{10}$$

Then the feature sequence F_i^k processed by K-layers of STL will be input into the convolution block at the end of DCSTB module for fusion. Each DCSTB can be regarded as a short-term memory module, and the convolution block at the end adaptively gives different weights to each Swin-Transformer in the DCSTB module to fuse the output of different Swin-Transformer layers, and determines how much short-term memory the DCSTB will retain. The fused features need to be added to the input

feature F_i^0,to strengthen the long-term memory of the network. The calculation formula of the above process is,

$$\mathrm{F_{i,out}} = \mathrm{H_{conv_i}}\left(\mathrm{F_i^1}, \mathrm{F_i^2}, \ldots, \mathrm{F_i^k}\right) + \mathrm{F_i^0}, \tag{11}$$

Among them, $F_{i,out}$is the output of the i_{th} DCSTB module, and H_{conv_i} represents the convolution block in the i_{th} DCSTB module.

Each DCSTB can be regarded as a short-term memory module. The convolution layer at the end of each DCSTB adaptively gives different weights to each Swin-Transformer to fuse the output of different Swin-Transformer layers, and determines how much short-term memory will be retained. Specifically, there are two reasons for adding a convolution layer at the end of each DCSTB module. The first reason is that the last convolutional layer in each DCSTB module can adaptively assign a fusion weight to the residual connections from each sub-layer in DCSTB module, which is equivalent to adding a gated fusion layer at the end of each DCSTB module. The second reason is to convert 1D features into 2D features and perform preliminary processing so that they can be fed into the final gated fusion (also implemented by the convolution layer) for weighted fusion.

3. Loss Function

We designed a hybrid loss function consisting of $L_1 - norm$ loss, perceptual loss and SSIM loss, to train the Gated Fusion Transformer network. The $L_1 - norm$ loss calculates the absolute distance between two images as $\mathrm{Loss_{L_1}(I_R, I_G)} = \left|\left|\mathrm{I_R} - \mathrm{I_G}\right|\right|_{\mathrm{L_1}}$, where I_R represents the reconstructed image by the network, and I_G denotes its ground truth. The perceptual loss is defined as the $L_2 - norm$ distance between feature maps output by the pool-3 layer of a VGG19 network pretrained on ImageNet as $\mathrm{Loss_{PER}(I_R, I_G)} = \left|\left|\varphi(\mathrm{I_R}) - \varphi(\mathrm{I_G})\right|\right|_{\mathrm{L_2}}$, where the $\varphi(\cdot)$ operation extracts feature maps. The perceptual loss regulates different-frequency similarity in the feature space. The SSIM loss is calculated as $\mathrm{Loss_{SSIM}} = 1 - \mathrm{SSIM(I_R, I_G)}$, which further regulates the two images' similarity in the structral domain. To sum, the loss function for network training is

$$\begin{gathered}\mathrm{Loss}\left(\mathrm{I_{RHQ}}, \mathrm{I_{HQ}}\right) = \alpha \mathrm{Loss_{L_1}}\left(\mathrm{I_{RHQ}}, \mathrm{I_{HQ}}\right) \\ +\beta \mathrm{Loss_{PER}}\left(\mathrm{I_{RHQ}}, \mathrm{I_{HQ}}\right) + \gamma \mathrm{Loss_{SSIM}}\left(\mathrm{I_{RHQ}}, \mathrm{I_{HQ}}\right),\end{gathered} \tag{12}$$

where α, β and γ are hyperparameters balancing the three loss parts. In our implementation, these hyperparameters were set as $\alpha = 0.1, \beta = 10$ and $\gamma = 100$ after careful network tuning.

Supplementary Note 11 - Experiment settings and specifications.

In this work, we conducted various experiments with different settings and specifications. To facilitate easy reference for readers, we have created a supplementary table that provides a comprehensive overview of the different experiment settings and specifications conducted in this work.

For the USAF Resolution target / PCB board and Microfluidic inspection experiments, we varied the bit depth from 1 to 10 bits. Besides, we applied super-resolution techniques at the scale factors of ×2 and ×4 to enhance the solution.

Regarding the single-photon FPM (Fourier Ptychographic Microscopy) experiments, the input images had a resolution of 32×32 pixels with an exposure time of 3μs. Through the implementation of FPM, we achieved a higher resolution of 192×192 pixels, which was subsequently further enhanced to 384×384 pixels.

In the high-speed single-photon imaging experiments, we varied the bit depth from 1 to 10 bits. Additionally, we employed super-resolution techniques at the scale factors of ×2 and ×4 to further enhance the results.

Supplementary Table 2: Settings and Specifications of Different Experiments

Experiment	Figure	Light source	Image size/pixels ×pixels	Enhanced size/pixels×pixels	Bit depth
Large-scale imaging	1	488nm laser power	64×32	128×64/ 256×128	1~10 bit
USAF Resolution target/ PCB board imaging	2	Olympus BX53	64×32	128×64/ 256×128	1~10 bit
Microfluidic inspection	3	Cnoptec Microscopy	64×32	128×64	1~10 bit
Single-photon FPM	4	Adafruit P4 LED array	32×32	384×384	7 bit
High-speed single-photon imaging	5	GCI-060411 LED array	64×32	128×64/ 256×128	1~10 bit

Supplementary References

1. Everingham, M. and Van~Gool, L. and Williams, C. K. I. and Winn, J. and Zisserman, A. The PASCAL Visual Object Classes Challebge 2007 (VOC2007) Results,http://www.pascal-network.org/challenges/VOC/voc2007/workshop/index.html
2. Everingham, M. and Van~Gool, L. and Williams, C. K. I. and Winn, J. and Zisserman, A. The PASCAL Visual Object Classes Challebge 2012 (VOC2012) Results,http://www.pascal-network.org/challenges/VOC/voc2012/workshop/index.html
3. F. Zhuang et al. "A Comprehensive Survey on Transfer Learning," in Proceedings of the IEEE, vol. 109, no. 1, pp. 43-76, Jan. 2021, doi: 10.1109/JPROC.2020.3004555.
