# Supplementary material for: High-resolution single-photon imaging with physics-informed deep learning
Source: Nat Commun. 2023 Sep 22;14:5902. doi: 10.1038/s41467-023-41597-9 (PMC10516985; doi:10.1038/s41467-023-41597-9)
Supplement: Supplementary file 3 — Description of Additional Supplementary Files [file 41467_2023_41597_MOESM3_ESM.pdf]

Description of Additional Supplementary Files

Title: **Supplementary Movie 1**

Description: High-speed single-photon imaging demonstration video
